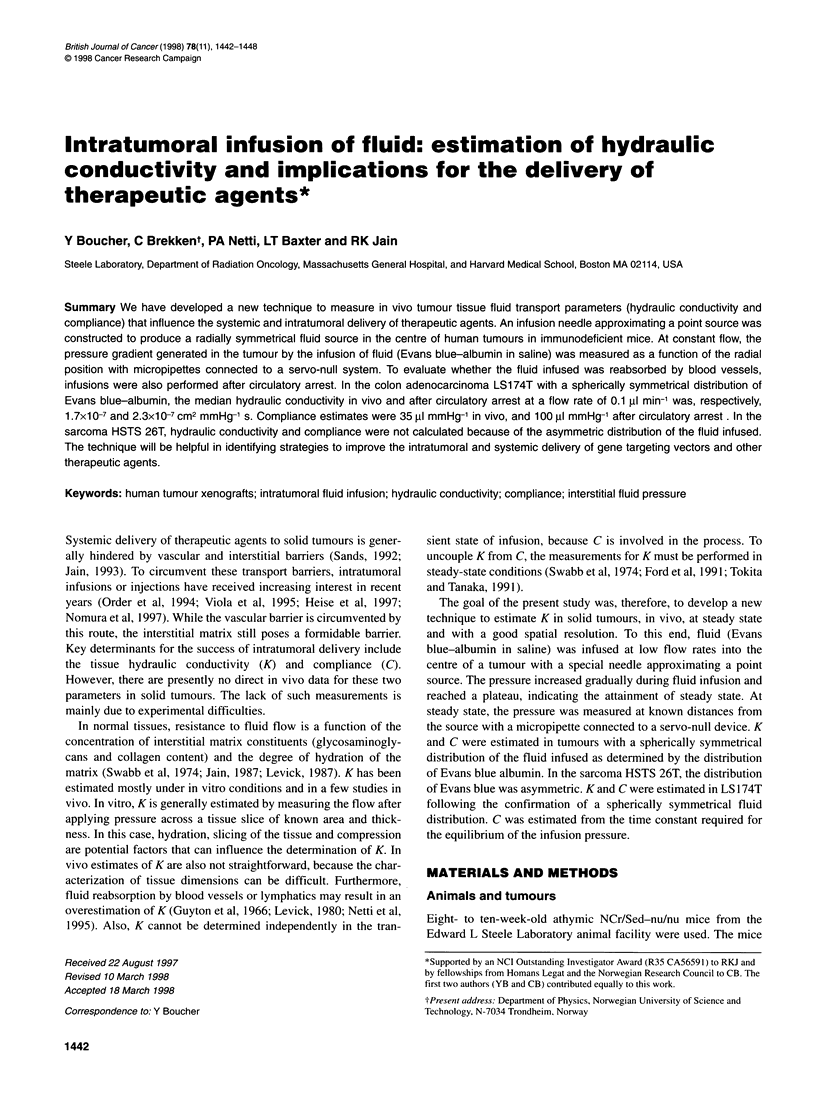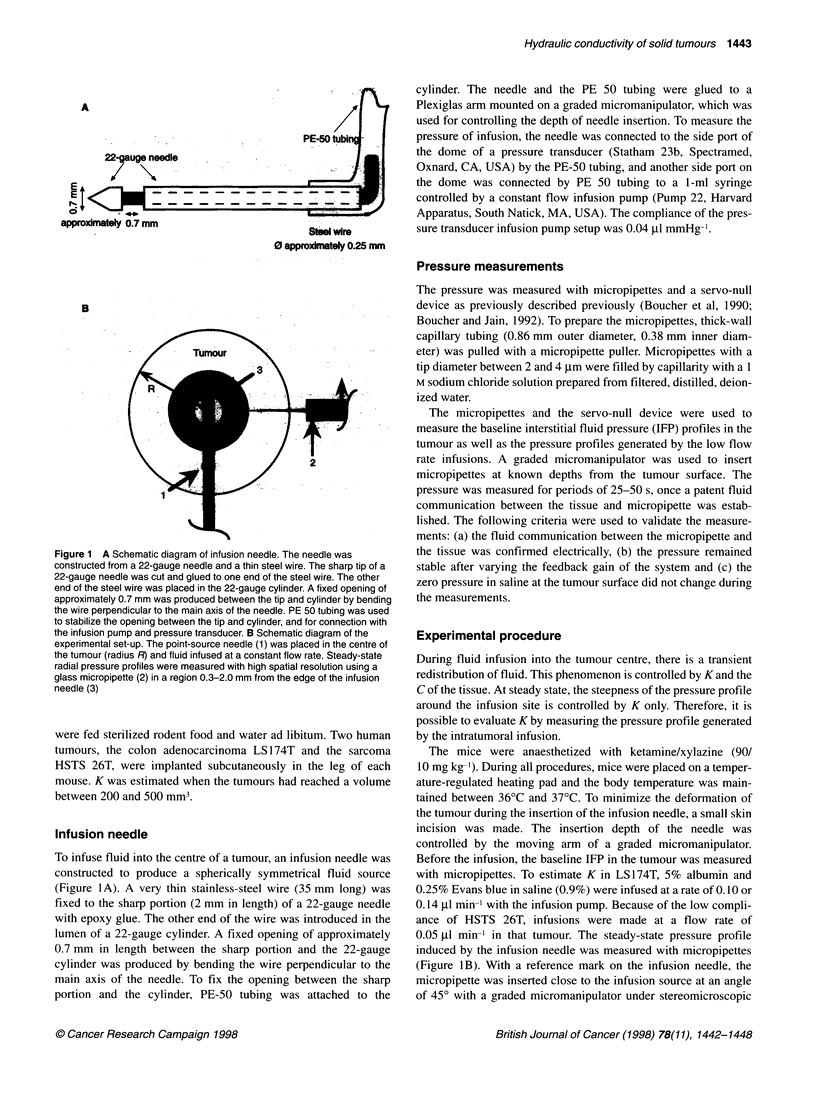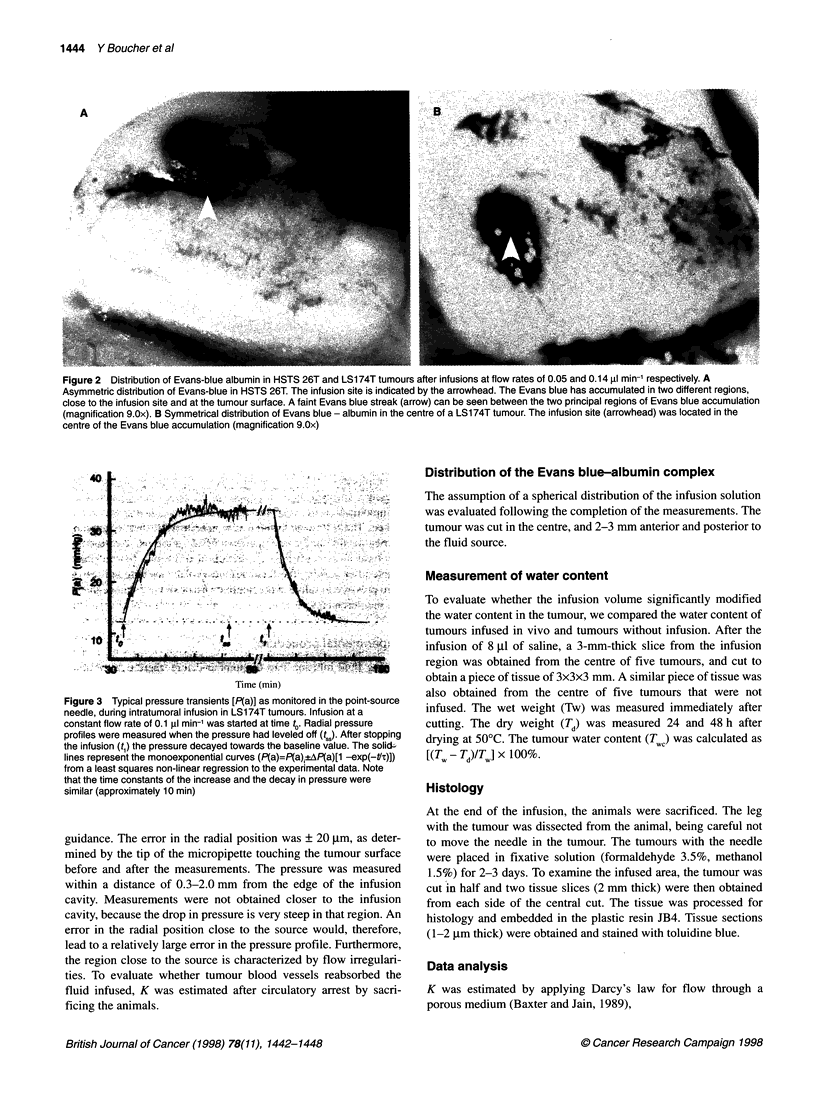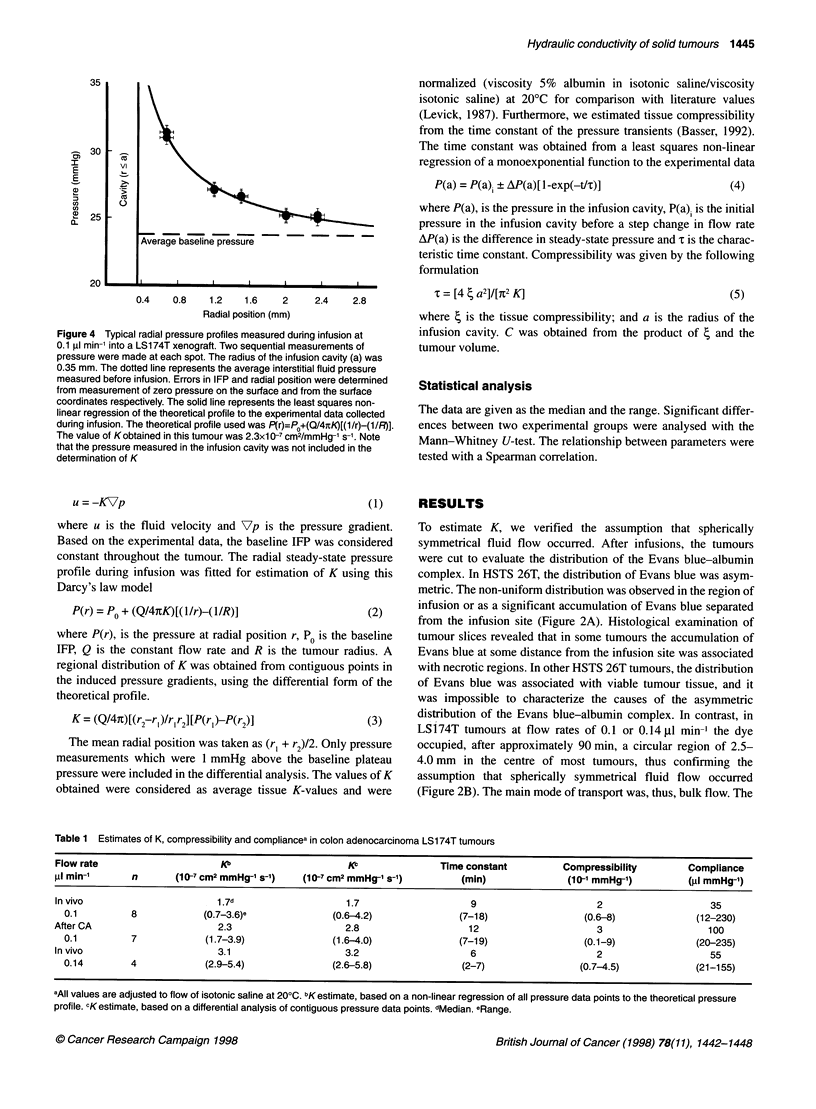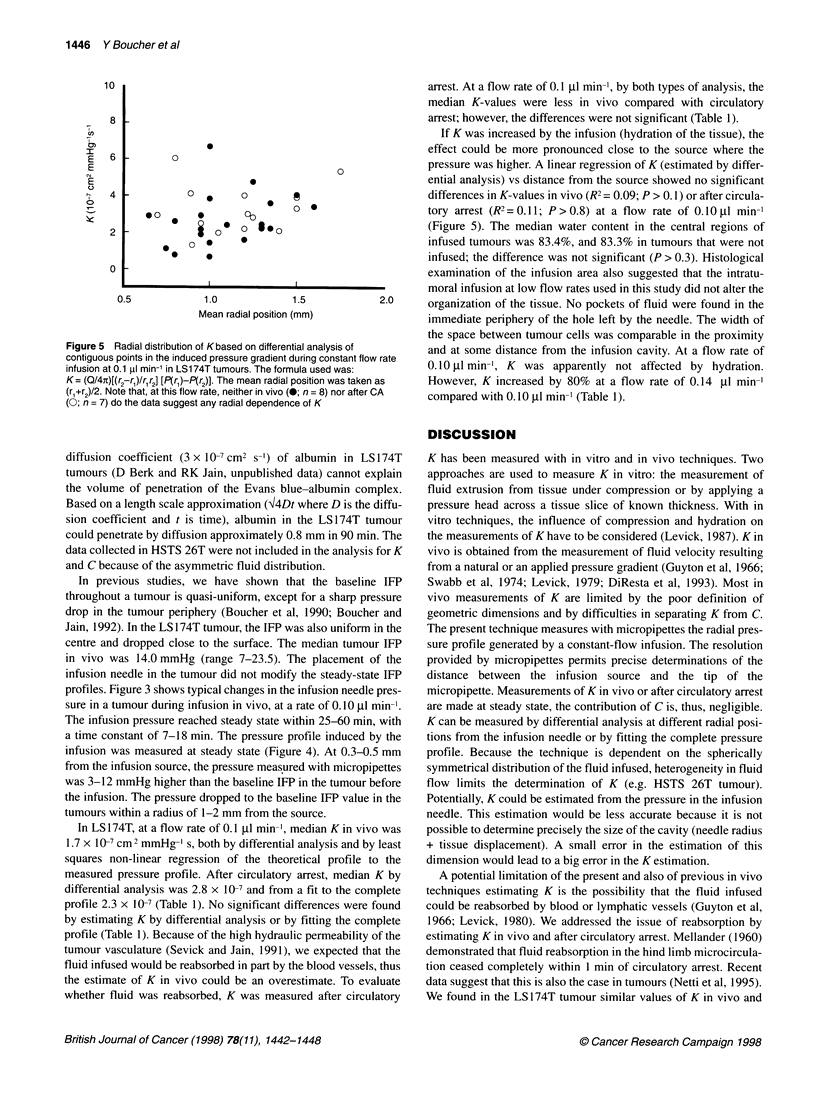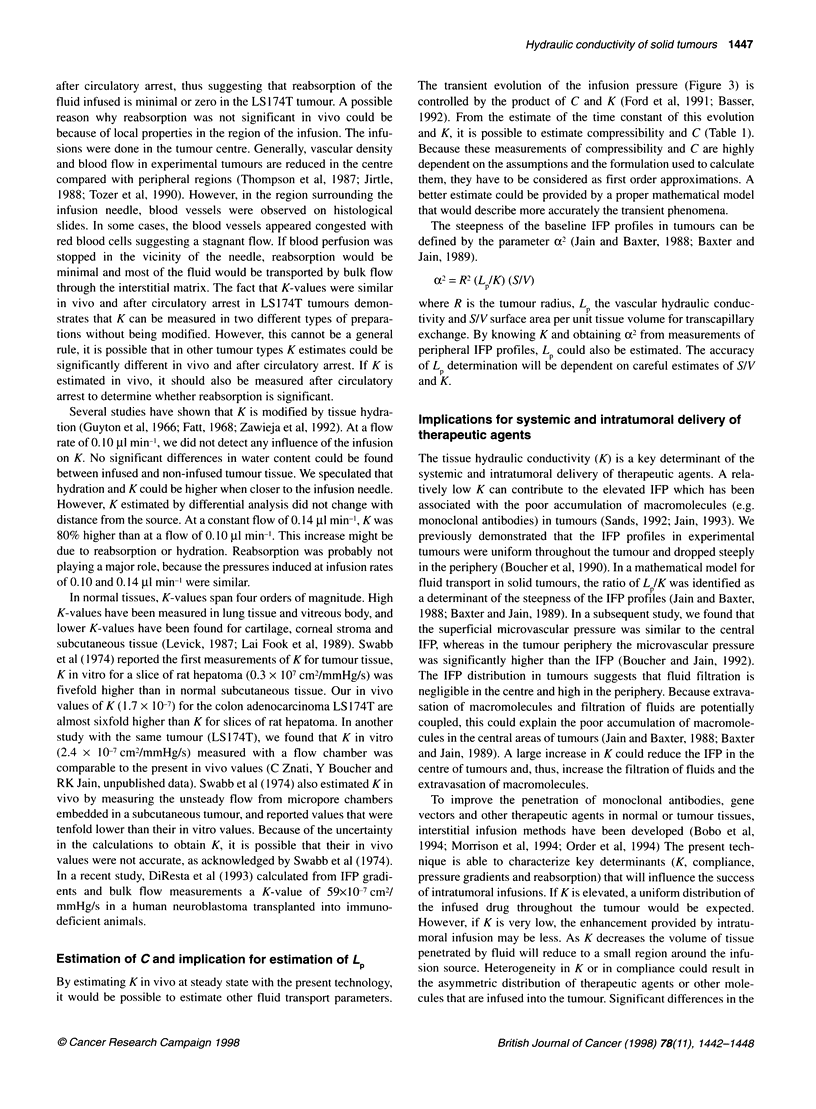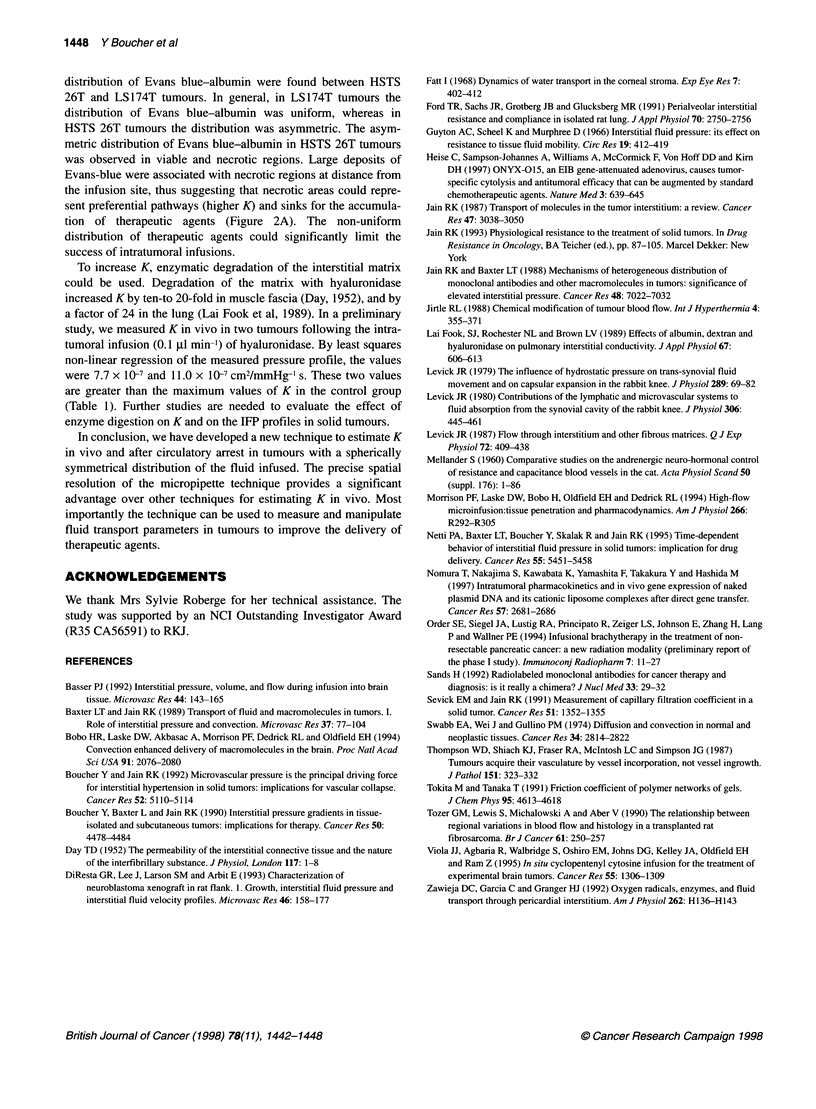# Intratumoral infusion of fluid: estimation of hydraulic conductivity and implications for the delivery of therapeutic agents.

**DOI:** 10.1038/bjc.1998.705

**Published:** 1998-12

**Authors:** Y. Boucher, C. Brekken, P. A. Netti, L. T. Baxter, R. K. Jain

**Affiliations:** Department of Radiation Oncology, Massachusetts General Hospital, and Harvard Medical School, Boston 02114, USA.

## Abstract

**Images:**


British Journal of Cancer (1998) 78(11), 1442–1448
© 1998 Cancer Research Campaign

### Intratumoral infusion of fluid: estimation of hydraulic conductivity and implications for the delivery of therapeutic agents*

**Y Boucher, C Brekken†, PA Netti, LT Baxter and RK Jain**

Steele Laboratory, Department of Radiation Oncology, Massachusetts General Hospital, and Harvard Medical School, Boston MA 02114, USA

**Summary** We have developed a new technique to measure in vivo tumour tissue fluid transport parameters (hydraulic conductivity and compliance) that influence the systemic and intratumoral delivery of therapeutic agents. An infusion needle approximating a point source was constructed to produce a radially symmetrical fluid source in the centre of human tumours in immunodeficient mice. At constant flow, the pressure gradient generated in the tumour by the infusion of fluid (Evans blue–albumin in saline) was measured as a function of the radial position with micropipettes connected to a servo-null system. To evaluate whether the fluid infused was reabsorbed by blood vessels, infusions were also performed after circulatory arrest. In the colon adenocarcinoma LS174T with a spherically symmetrical distribution of Evans blue–albumin, the median hydraulic conductivity in vivo and after circulatory arrest at a flow rate of 0.1 μl $min^{-1}$ was, respectively, $1.7 \times 10^{-7}$ and $2.3 \times 10^{-7}$ $cm^2$ $mmHg^{-1}$ s. Compliance estimates were 35 μl $mmHg^{-1}$ in vivo, and 100 μl $mmHg^{-1}$ after circulatory arrest . In the sarcoma HSTS 26T, hydraulic conductivity and compliance were not calculated because of the asymmetric distribution of the fluid infused. The technique will be helpful in identifying strategies to improve the intratumoral and systemic delivery of gene targeting vectors and other therapeutic agents.

**Keywords:** human tumour xenografts; intratumoral fluid infusion; hydraulic conductivity; compliance; interstitial fluid pressure

Systemic delivery of therapeutic agents to solid tumours is generally hindered by vascular and interstitial barriers (Sands, 1992; Jain, 1993). To circumvent these transport barriers, intratumoral infusions or injections have received increasing interest in recent years (Order et al, 1994; Viola et al, 1995; Heise et al, 1997; Nomura et al, 1997). While the vascular barrier is circumvented by this route, the interstitial matrix still poses a formidable barrier. Key determinants for the success of intratumoral delivery include the tissue hydraulic conductivity ($K$) and compliance ($C$). However, there are presently no direct in vivo data for these two parameters in solid tumours. The lack of such measurements is mainly due to experimental difficulties.

In normal tissues, resistance to fluid flow is a function of the concentration of interstitial matrix constituents (glycosaminoglycans and collagen content) and the degree of hydration of the matrix (Swabb et al, 1974; Jain, 1987; Levick, 1987). $K$ has been estimated mostly under in vitro conditions and in a few studies in vivo. In vitro, $K$ is generally estimated by measuring the flow after applying pressure across a tissue slice of known area and thickness. In this case, hydration, slicing of the tissue and compression are potential factors that can influence the determination of $K$. In vivo estimates of $K$ are also not straightforward, because the characterization of tissue dimensions can be difficult. Furthermore, fluid reabsorption by blood vessels or lymphatics may result in an overestimation of $K$ (Guyton et al, 1966; Levick, 1980; Netti et al, 1995). Also, $K$ cannot be determined independently in the transient state of infusion, because $C$ is involved in the process. To uncouple $K$ from $C$, the measurements for $K$ must be performed in steady-state conditions (Swabb et al, 1974; Ford et al, 1991; Tokita and Tanaka, 1991).

The goal of the present study was, therefore, to develop a new technique to estimate $K$ in solid tumours, in vivo, at steady state and with a good spatial resolution. To this end, fluid (Evans blue-albumin in saline) was infused at low flow rates into the centre of a tumour with a special needle approximating a point source. The pressure increased gradually during fluid infusion and reached a plateau, indicating the attainment of steady state. At steady state, the pressure was measured at known distances from the source with a micropipette connected to a servo-null device. $K$ and $C$ were estimated in tumours with a spherically symmetrical distribution of the fluid infused as determined by the distribution of Evans blue albumin. In the sarcoma HSTS 26T, the distribution of Evans blue was asymmetric. $K$ and $C$ were estimated in LS174T following the confirmation of a spherically symmetrical fluid distribution. $C$ was estimated from the time constant required for the equilibrium of the infusion pressure.

### MATERIALS AND METHODS

### Animals and tumours

Eight- to ten-week-old athymic NCr/Sed–nu/nu mice from the Edward L Steele Laboratory animal facility were used. The mice

Received 22 August 1997
Revised 10 March 1998
Accepted 18 March 1998

Correspondence to: Y Boucher

*Supported by an NCI Outstanding Investigator Award (R35 CA56591) to RKJ and by fellowships from Homans Legat and the Norwegian Research Council to CB. The first two authors (YB and CB) contributed equally to this work.

†Present address: Department of Physics, Norwegian University of Science and Technology, N-7034 Trondheim, Norway

**Figure 1** **A** Schematic diagram of infusion needle. The needle was constructed from a 22-gauge needle and a thin steel wire. The sharp tip of a 22-gauge needle was cut and glued to one end of the steel wire. The other end of the steel wire was placed in the 22-gauge cylinder. A fixed opening of approximately 0.7 mm was produced between the tip and cylinder by bending the wire perpendicular to the main axis of the needle. PE 50 tubing was used to stabilize the opening between the tip and cylinder, and for connection with the infusion pump and pressure transducer. **B** Schematic diagram of the experimental set-up. The point-source needle (1) was placed in the centre of the tumour (radius *R*) and fluid infused at a constant flow rate. Steady-state radial pressure profiles were measured with high spatial resolution using a glass micropipette (2) in a region 0.3–2.0 mm from the edge of the infusion needle (3)

were fed sterilized rodent food and water ad libitum. Two human tumours, the colon adenocarcinoma LS174T and the sarcoma HSTS 26T, were implanted subcutaneously in the leg of each mouse. *K* was estimated when the tumours had reached a volume between 200 and 500 $mm^3$.

### Infusion needle

To infuse fluid into the centre of a tumour, an infusion needle was constructed to produce a spherically symmetrical fluid source (Figure 1A). A very thin stainless-steel wire (35 mm long) was fixed to the sharp portion (2 mm in length) of a 22-gauge needle with epoxy glue. The other end of the wire was introduced in the lumen of a 22-gauge cylinder. A fixed opening of approximately 0.7 mm in length between the sharp portion and the 22-gauge cylinder was produced by bending the wire perpendicular to the main axis of the needle. To fix the opening between the sharp portion and the cylinder, PE-50 tubing was attached to the cylinder. The needle and the PE 50 tubing were glued to a Plexiglas arm mounted on a graded micromanipulator, which was used for controlling the depth of needle insertion. To measure the pressure of infusion, the needle was connected to the side port of the dome of a pressure transducer (Statham 23b, Spectramed, Oxnard, CA, USA) by the PE-50 tubing, and another side port on the dome was connected by PE 50 tubing to a 1-ml syringe controlled by a constant flow infusion pump (Pump 22, Harvard Apparatus, South Natick, MA, USA). The compliance of the pressure transducer infusion pump setup was 0.04 $\mu l\ mmHg^{-1}$.

### Pressure measurements

The pressure was measured with micropipettes and a servo-null device as previously described previously (Boucher et al, 1990; Boucher and Jain, 1992). To prepare the micropipettes, thick-wall capillary tubing (0.86 mm outer diameter, 0.38 mm inner diameter) was pulled with a micropipette puller. Micropipettes with a tip diameter between 2 and 4 μm were filled by capillarity with a 1 M sodium chloride solution prepared from filtered, distilled, deionized water.

The micropipettes and the servo-null device were used to measure the baseline interstitial fluid pressure (IFP) profiles in the tumour as well as the pressure profiles generated by the low flow rate infusions. A graded micromanipulator was used to insert micropipettes at known depths from the tumour surface. The pressure was measured for periods of 25–50 s, once a patent fluid communication between the tissue and micropipette was established. The following criteria were used to validate the measurements: (a) the fluid communication between the micropipette and the tissue was confirmed electrically, (b) the pressure remained stable after varying the feedback gain of the system and (c) the zero pressure in saline at the tumour surface did not change during the measurements.

### Experimental procedure

During fluid infusion into the tumour centre, there is a transient redistribution of fluid. This phenomenon is controlled by *K* and the *C* of the tissue. At steady state, the steepness of the pressure profile around the infusion site is controlled by *K* only. Therefore, it is possible to evaluate *K* by measuring the pressure profile generated by the intratumoral infusion.

The mice were anaesthetized with ketamine/xylazine (90/10 $mg\ kg^{-1}$). During all procedures, mice were placed on a temperature-regulated heating pad and the body temperature was maintained between 36°C and 37°C. To minimize the deformation of the tumour during the insertion of the infusion needle, a small skin incision was made. The insertion depth of the needle was controlled by the moving arm of a graded micromanipulator. Before the infusion, the baseline IFP in the tumour was measured with micropipettes. To estimate *K* in LS174T, 5% albumin and 0.25% Evans blue in saline (0.9%) were infused at a rate of 0.10 or 0.14 $\mu l\ min^{-1}$ with the infusion pump. Because of the low compliance of HSTS 26T, infusions were made at a flow rate of 0.05 $\mu l\ min^{-1}$ in that tumour. The steady-state pressure profile induced by the infusion needle was measured with micropipettes (Figure 1B). With a reference mark on the infusion needle, the micropipette was inserted close to the infusion source at an angle of 45° with a graded micromanipulator under stereomicroscopic

**Figure 2** Distribution of Evans-blue albumin in HSTS 26T and LS174T tumours after infusions at flow rates of 0.05 and 0.14 μl min⁻¹ respectively. **A** Asymmetric distribution of Evans-blue in HSTS 26T. The infusion site is indicated by the arrowhead. The Evans blue has accumulated in two different regions, close to the infusion site and at the tumour surface. A faint Evans blue streak (arrow) can be seen between the two principal regions of Evans blue accumulation (magnification 9.0×). **B** Symmetrical distribution of Evans blue – albumin in the centre of a LS174T tumour. The infusion site (arrowhead) was located in the centre of the Evans blue accumulation (magnification 9.0×)

**Figure 3** Typical pressure transients [$P$(a)] as monitored in the point-source needle, during intratumoral infusion in LS174T tumours. Infusion at a constant flow rate of 0.1 μl min⁻¹ was started at time $t_0$. Radial pressure profiles were measured when the pressure had leveled off ($t_{ss}$). After stopping the infusion ($t_1$) the pressure decayed towards the baseline value. The solid lines represent the monoexponential curves ($P(a)=P(a)_i \pm \Delta P(a)[1 - \exp(-t/\tau)]$) from a least squares non-linear regression to the experimental data. Note that the time constants of the increase and the decay in pressure were similar (approximately 10 min)

guidance. The error in the radial position was ± 20 μm, as determined by the tip of the micropipette touching the tumour surface before and after the measurements. The pressure was measured within a distance of 0.3–2.0 mm from the edge of the infusion cavity. Measurements were not obtained closer to the infusion cavity, because the drop in pressure is very steep in that region. An error in the radial position close to the source would, therefore, lead to a relatively large error in the pressure profile. Furthermore, the region close to the source is characterized by flow irregularities. To evaluate whether tumour blood vessels reabsorbed the fluid infused, $K$ was estimated after circulatory arrest by sacrificing the animals.

### Distribution of the Evans blue–albumin complex

The assumption of a spherical distribution of the infusion solution was evaluated following the completion of the measurements. The tumour was cut in the centre, and 2–3 mm anterior and posterior to the fluid source.

### Measurement of water content

To evaluate whether the infusion volume significantly modified the water content in the tumour, we compared the water content of tumours infused in vivo and tumours without infusion. After the infusion of 8 μl of saline, a 3-mm-thick slice from the infusion region was obtained from the centre of five tumours, and cut to obtain a piece of tissue of 3×3×3 mm. A similar piece of tissue was also obtained from the centre of five tumours that were not infused. The wet weight (Tw) was measured immediately after cutting. The dry weight ($T_d$) was measured 24 and 48 h after drying at 50°C. The tumour water content ($T_{wc}$) was calculated as $[(T_w - T_d)/T_w] \times 100\%$.

### Histology

At the end of the infusion, the animals were sacrificed. The leg with the tumour was dissected from the animal, being careful not to move the needle in the tumour. The tumours with the needle were placed in fixative solution (formaldehyde 3.5%, methanol 1.5%) for 2–3 days. To examine the infused area, the tumour was cut in half and two tissue slices (2 mm thick) were then obtained from each side of the central cut. The tissue was processed for histology and embedded in the plastic resin JB4. Tissue sections (1–2 μm thick) were obtained and stained with toluidine blue.

### Data analysis

$K$ was estimated by applying Darcy's law for flow through a porous medium (Baxter and Jain, 1989),

 © Cancer Research Campaign 1998

**Figure 4** Typical radial pressure profiles measured during infusion at 0.1 μl min$^{-1}$ into a LS174T xenograft. Two sequential measurements of pressure were made at each spot. The radius of the infusion cavity (a) was 0.35 mm. The dotted line represents the average interstitial fluid pressure measured before infusion. Errors in IFP and radial position were determined from measurement of zero pressure on the surface and from the surface coordinates respectively. The solid line represents the least squares non-linear regression of the theoretical profile to the experimental data collected during infusion. The theoretical profile used was $P(r)=P_0+(Q/4\pi K)[(1/r)-(1/R)]$. The value of $K$ obtained in this tumour was 2.3×10$^{-7}$ cm$^2$/mmHg$^{-1}$ s$^{-1}$. Note that the pressure measured in the infusion cavity was not included in the determination of $K$

$$u = -K\nabla p \quad (1)$$

where $u$ is the fluid velocity and $\nabla p$ is the pressure gradient. Based on the experimental data, the baseline IFP was considered constant throughout the tumour. The radial steady-state pressure profile during infusion was fitted for estimation of $K$ using this Darcy's law model

$$P(r) = P_0 + (Q/4\pi K)[(1/r)-(1/R)] \quad (2)$$

where $P(r)$, is the pressure at radial position $r$, $P_0$ is the baseline IFP, $Q$ is the constant flow rate and $R$ is the tumour radius. A regional distribution of $K$ was obtained from contiguous points in the induced pressure gradients, using the differential form of the theoretical profile.

$$K = (Q/4\pi)[(r_2-r_1)/r_1r_2][P(r_1)-P(r_2)] \quad (3)$$

The mean radial position was taken as $(r_1 + r_2)/2$. Only pressure measurements which were 1 mmHg above the baseline plateau pressure were included in the differential analysis. The values of $K$ obtained were considered as average tissue $K$-values and were normalized (viscosity 5% albumin in isotonic saline/viscosity isotonic saline) at 20°C for comparison with literature values (Levick, 1987). Furthermore, we estimated tissue compressibility from the time constant of the pressure transients (Basser, 1992). The time constant was obtained from a least squares non-linear regression of a monoexponential function to the experimental data

$$P(\mathrm{a}) = P(\mathrm{a})_i \pm \Delta P(\mathrm{a})[1-\exp(-t/\tau)] \quad (4)$$

where $P(\mathrm{a})$, is the pressure in the infusion cavity, $P(\mathrm{a})_i$ is the initial pressure in the infusion cavity before a step change in flow rate $\Delta P(\mathrm{a})$ is the difference in steady-state pressure and $\tau$ is the characteristic time constant. Compressibility was given by the following formulation

$$\tau = [4\,\xi\, a^2]/[\pi^2 K] \quad (5)$$

where $\xi$ is the tissue compressibility; and $a$ is the radius of the infusion cavity. $C$ was obtained from the product of $\xi$ and the tumour volume.

### Statistical analysis

The data are given as the median and the range. Significant differences between two experimental groups were analysed with the Mann–Whitney $U$-test. The relationship between parameters were tested with a Spearman correlation.

### RESULTS

To estimate $K$, we verified the assumption that spherically symmetrical fluid flow occurred. After infusions, the tumours were cut to evaluate the distribution of the Evans blue–albumin complex. In HSTS 26T, the distribution of Evans blue was asymmetric. The non-uniform distribution was observed in the region of infusion or as a significant accumulation of Evans blue separated from the infusion site (Figure 2A). Histological examination of tumour slices revealed that in some tumours the accumulation of Evans blue at some distance from the infusion site was associated with necrotic regions. In other HSTS 26T tumours, the distribution of Evans blue was associated with viable tumour tissue, and it was impossible to characterize the causes of the asymmetric distribution of the Evans blue–albumin complex. In contrast, in LS174T tumours at flow rates of 0.1 or 0.14 μl min$^{-1}$ the dye occupied, after approximately 90 min, a circular region of 2.5–4.0 mm in the centre of most tumours, thus confirming the assumption that spherically symmetrical fluid flow occurred (Figure 2B). The main mode of transport was, thus, bulk flow. The

**Table 1** Estimates of K, compressibility and compliance[a] in colon adenocarcinoma LS174T tumours

| Flow rate μl min$^{-1}$ | $n$ | $K$[b] (10$^{-7}$ cm$^2$ mmHg$^{-1}$ s$^{-1}$) | $K$[c] (10$^{-7}$ cm$^2$ mmHg$^{-1}$ s$^{-1}$) | Time constant (min) | Compressibility (10$^{-1}$ mmHg$^{-1}$) | Compliance (μl mmHg$^{-1}$) |
|---|---|---|---|---|---|---|
| In vivo | | 1.7[d] | 1.7 | 9 | 2 | 35 |
| 0.1 | 8 | (0.7–3.6)[e] | (0.6–4.2) | (7–18) | (0.6–8) | (12–230) |
| After CA | | 2.3 | 2.8 | 12 | 3 | 100 |
| 0.1 | 7 | (1.7–3.9) | (1.6–4.0) | (7–19) | (0.1–9) | (20–235) |
| In vivo | | 3.1 | 3.2 | 6 | 2 | 55 |
| 0.14 | 4 | (2.9–5.4) | (2.6–5.8) | (2–7) | (0.7–4.5) | (21–155) |

[a]All values are adjusted to flow of isotonic saline at 20°C. [b]$K$ estimate, based on a non-linear regression of all pressure data points to the theoretical pressure profile. [c]$K$ estimate, based on a differential analysis of contiguous pressure data points. [d]Median. [e]Range.

© Cancer Research Campaign 1998 

**Figure 5** Radial distribution of $K$ based on differential analysis of contiguous points in the induced pressure gradient during constant flow rate infusion at 0.1 µl min⁻¹ in LS174T tumours. The formula used was: $K = (Q/4\pi)[(r_2-r_1)/r_1r_2]\,[P(r_1)-P(r_2)]$. The mean radial position was taken as $(r_1+r_2)/2$. Note that, at this flow rate, neither in vivo (●; $n$ = 8) nor after CA (○; $n$ = 7) do the data suggest any radial dependence of $K$

diffusion coefficient ($3 \times 10^{-7}$ cm² s⁻¹) of albumin in LS174T tumours (D Berk and RK Jain, unpublished data) cannot explain the volume of penetration of the Evans blue–albumin complex. Based on a length scale approximation ($\sqrt{4Dt}$ where $D$ is the diffusion coefficient and $t$ is time), albumin in the LS174T tumour could penetrate by diffusion approximately 0.8 mm in 90 min. The data collected in HSTS 26T were not included in the analysis for $K$ and $C$ because of the asymmetric fluid distribution.

In previous studies, we have shown that the baseline IFP throughout a tumour is quasi-uniform, except for a sharp pressure drop in the tumour periphery (Boucher et al, 1990; Boucher and Jain, 1992). In the LS174T tumour, the IFP was also uniform in the centre and dropped close to the surface. The median tumour IFP in vivo was 14.0 mmHg (range 7–23.5). The placement of the infusion needle in the tumour did not modify the steady-state IFP profiles. Figure 3 shows typical changes in the infusion needle pressure in a tumour during infusion in vivo, at a rate of 0.10 µl min⁻¹. The infusion pressure reached steady state within 25–60 min, with a time constant of 7–18 min. The pressure profile induced by the infusion was measured at steady state (Figure 4). At 0.3–0.5 mm from the infusion source, the pressure measured with micropipettes was 3–12 mmHg higher than the baseline IFP in the tumour before the infusion. The pressure dropped to the baseline IFP value in the tumours within a radius of 1–2 mm from the source.

In LS174T, at a flow rate of 0.1 µl min⁻¹, median $K$ in vivo was $1.7 \times 10^{-7}$ cm² mmHg⁻¹ s, both by differential analysis and by least squares non-linear regression of the theoretical profile to the measured pressure profile. After circulatory arrest, median $K$ by differential analysis was $2.8 \times 10^{-7}$ and from a fit to the complete profile $2.3 \times 10^{-7}$ (Table 1). No significant differences were found by estimating $K$ by differential analysis or by fitting the complete profile (Table 1). Because of the high hydraulic permeability of the tumour vasculature (Sevick and Jain, 1991), we expected that the fluid infused would be reabsorbed in part by the blood vessels, thus the estimate of $K$ in vivo could be an overestimate. To evaluate whether fluid was reabsorbed, $K$ was measured after circulatory arrest. At a flow rate of 0.1 µl min⁻¹, by both types of analysis, the median $K$-values were less in vivo compared with circulatory arrest; however, the differences were not significant (Table 1).

If $K$ was increased by the infusion (hydration of the tissue), the effect could be more pronounced close to the source where the pressure was higher. A linear regression of $K$ (estimated by differential analysis) vs distance from the source showed no significant differences in $K$-values in vivo ($R^2 = 0.09$; $P > 0.1$) or after circulatory arrest ($R^2 = 0.11$; $P > 0.8$) at a flow rate of 0.10 µl min⁻¹ (Figure 5). The median water content in the central regions of infused tumours was 83.4%, and 83.3% in tumours that were not infused; the difference was not significant ($P > 0.3$). Histological examination of the infusion area also suggested that the intratumoral infusion at low flow rates used in this study did not alter the organization of the tissue. No pockets of fluid were found in the immediate periphery of the hole left by the needle. The width of the space between tumour cells was comparable in the proximity and at some distance from the infusion cavity. At a flow rate of 0.10 µl min⁻¹, $K$ was apparently not affected by hydration. However, $K$ increased by 80% at a flow rate of 0.14 µl min⁻¹ compared with 0.10 µl min⁻¹ (Table 1).

### DISCUSSION

$K$ has been measured with in vitro and in vivo techniques. Two approaches are used to measure $K$ in vitro: the measurement of fluid extrusion from tissue under compression or by applying a pressure head across a tissue slice of known thickness. With in vitro techniques, the influence of compression and hydration on the measurements of $K$ have to be considered (Levick, 1987). $K$ in vivo is obtained from the measurement of fluid velocity resulting from a natural or an applied pressure gradient (Guyton et al, 1966; Swabb et al, 1974; Levick, 1979; DiResta et al, 1993). Most in vivo measurements of $K$ are limited by the poor definition of geometric dimensions and by difficulties in separating $K$ from $C$. The present technique measures with micropipettes the radial pressure profile generated by a constant-flow infusion. The resolution provided by micropipettes permits precise determinations of the distance between the infusion source and the tip of the micropipette. Measurements of $K$ in vivo or after circulatory arrest are made at steady state, the contribution of $C$ is, thus, negligible. $K$ can be measured by differential analysis at different radial positions from the infusion needle or by fitting the complete pressure profile. Because the technique is dependent on the spherically symmetrical distribution of the fluid infused, heterogeneity in fluid flow limits the determination of $K$ (e.g. HSTS 26T tumour). Potentially, $K$ could be estimated from the pressure in the infusion needle. This estimation would be less accurate because it is not possible to determine precisely the size of the cavity (needle radius + tissue displacement). A small error in the estimation of this dimension would lead to a big error in the $K$ estimation.

A potential limitation of the present and also of previous in vivo techniques estimating $K$ is the possibility that the fluid infused could be reabsorbed by blood or lymphatic vessels (Guyton et al, 1966; Levick, 1980). We addressed the issue of reabsorption by estimating $K$ in vivo and after circulatory arrest. Mellander (1960) demonstrated that fluid reabsorption in the hind limb microcirculation ceased completely within 1 min of circulatory arrest. Recent data suggest that this is also the case in tumours (Netti et al, 1995). We found in the LS174T tumour similar values of $K$ in vivo and

 © Cancer Research Campaign 1998

after circulatory arrest, thus suggesting that reabsorption of the fluid infused is minimal or zero in the LS174T tumour. A possible reason why reabsorption was not significant in vivo could be because of local properties in the region of the infusion. The infusions were done in the tumour centre. Generally, vascular density and blood flow in experimental tumours are reduced in the centre compared with peripheral regions (Thompson et al, 1987; Jirtle, 1988; Tozer et al, 1990). However, in the region surrounding the infusion needle, blood vessels were observed on histological slides. In some cases, the blood vessels appeared congested with red blood cells suggesting a stagnant flow. If blood perfusion was stopped in the vicinity of the needle, reabsorption would be minimal and most of the fluid would be transported by bulk flow through the interstitial matrix. The fact that *K*-values were similar in vivo and after circulatory arrest in LS174T tumours demonstrates that *K* can be measured in two different types of preparations without being modified. However, this cannot be a general rule, it is possible that in other tumour types *K* estimates could be significantly different in vivo and after circulatory arrest. If *K* is estimated in vivo, it should also be measured after circulatory arrest to determine whether reabsorption is significant.

Several studies have shown that *K* is modified by tissue hydration (Guyton et al, 1966; Fatt, 1968; Zawieja et al, 1992). At a flow rate of 0.10 μl $min^{-1}$, we did not detect any influence of the infusion on *K*. No significant differences in water content could be found between infused and non-infused tumour tissue. We speculated that hydration and *K* could be higher when closer to the infusion needle. However, *K* estimated by differential analysis did not change with distance from the source. At a constant flow of 0.14 μl $min^{-1}$, *K* was 80% higher than at a flow of 0.10 μl $min^{-1}$. This increase might be due to reabsorption or hydration. Reabsorption was probably not playing a major role, because the pressures induced at infusion rates of 0.10 and 0.14 μl $min^{-1}$ were similar.

In normal tissues, *K*-values span four orders of magnitude. High *K*-values have been measured in lung tissue and vitreous body, and lower *K*-values have been found for cartilage, corneal stroma and subcutaneous tissue (Levick, 1987; Lai Fook et al, 1989). Swabb et al (1974) reported the first measurements of *K* for tumour tissue, *K* in vitro for a slice of rat hepatoma ($0.3 \times 10^7$ $cm^2$/mmHg/s) was fivefold higher than in normal subcutaneous tissue. Our in vivo values of *K* ($1.7 \times 10^{-7}$) for the colon adenocarcinoma LS174T are almost sixfold higher than *K* for slices of rat hepatoma. In another study with the same tumour (LS174T), we found that *K* in vitro ($2.4 \times 10^{-7}$ $cm^2$/mmHg/s) measured with a flow chamber was comparable to the present in vivo values (C Znati, Y Boucher and RK Jain, unpublished data). Swabb et al (1974) also estimated *K* in vivo by measuring the unsteady flow from micropore chambers embedded in a subcutaneous tumour, and reported values that were tenfold lower than their in vitro values. Because of the uncertainty in the calculations to obtain *K*, it is possible that their in vivo values were not accurate, as acknowledged by Swabb et al (1974). In a recent study, DiResta et al (1993) calculated from IFP gradients and bulk flow measurements a *K*-value of $59 \times 10^{-7}$ $cm^2$/mmHg/s in a human neuroblastoma transplanted into immunodeficient animals.

### Estimation of *C* and implication for estimation of $L_p$

By estimating *K* in vivo at steady state with the present technology, it would be possible to estimate other fluid transport parameters. The transient evolution of the infusion pressure (Figure 3) is controlled by the product of *C* and *K* (Ford et al, 1991; Basser, 1992). From the estimate of the time constant of this evolution and *K*, it is possible to estimate compressibility and *C* (Table 1). Because these measurements of compressibility and *C* are highly dependent on the assumptions and the formulation used to calculate them, they have to be considered as first order approximations. A better estimate could be provided by a proper mathematical model that would describe more accurately the transient phenomena.

The steepness of the baseline IFP profiles in tumours can be defined by the parameter $\alpha^2$ (Jain and Baxter, 1988; Baxter and Jain, 1989).

$$\alpha^2 = R^2 (L_p/K) (S/V)$$

where *R* is the tumour radius, $L_p$ the vascular hydraulic conductivity and *S/V* surface area per unit tissue volume for transcapillary exchange. By knowing *K* and obtaining $\alpha^2$ from measurements of peripheral IFP profiles, $L_p$ could also be estimated. The accuracy of $L_p$ determination will be dependent on careful estimates of *S/V* and *K*.

### Implications for systemic and intratumoral delivery of therapeutic agents

The tissue hydraulic conductivity (*K*) is a key determinant of the systemic and intratumoral delivery of therapeutic agents. A relatively low *K* can contribute to the elevated IFP which has been associated with the poor accumulation of macromolecules (e.g. monoclonal antibodies) in tumours (Sands, 1992; Jain, 1993). We previously demonstrated that the IFP profiles in experimental tumours were uniform throughout the tumour and dropped steeply in the periphery (Boucher et al, 1990). In a mathematical model for fluid transport in solid tumours, the ratio of $L_p/K$ was identified as a determinant of the steepness of the IFP profiles (Jain and Baxter, 1988; Baxter and Jain, 1989). In a subsequent study, we found that the superficial microvascular pressure was similar to the central IFP, whereas in the tumour periphery the microvascular pressure was significantly higher than the IFP (Boucher and Jain, 1992). The IFP distribution in tumours suggests that fluid filtration is negligible in the centre and high in the periphery. Because extravasation of macromolecules and filtration of fluids are potentially coupled, this could explain the poor accumulation of macromolecules in the central areas of tumours (Jain and Baxter, 1988; Baxter and Jain, 1989). A large increase in *K* could reduce the IFP in the centre of tumours and, thus, increase the filtration of fluids and the extravasation of macromolecules.

To improve the penetration of monoclonal antibodies, gene vectors and other therapeutic agents in normal or tumour tissues, interstitial infusion methods have been developed (Bobo et al, 1994; Morrison et al, 1994; Order et al, 1994) The present technique is able to characterize key determinants (*K*, compliance, pressure gradients and reabsorption) that will influence the success of intratumoral infusions. If *K* is elevated, a uniform distribution of the infused drug throughout the tumour would be expected. However, if *K* is very low, the enhancement provided by intratumoral infusion may be less. As *K* decreases the volume of tissue penetrated by fluid will reduce to a small region around the infusion source. Heterogeneity in *K* or in compliance could result in the asymmetric distribution of therapeutic agents or other molecules that are infused into the tumour. Significant differences in the

© Cancer Research Campaign 1998

distribution of Evans blue–albumin were found between HSTS 26T and LS174T tumours. In general, in LS174T tumours the distribution of Evans blue–albumin was uniform, whereas in HSTS 26T tumours the distribution was asymmetric. The asymmetric distribution of Evans blue–albumin in HSTS 26T tumours was observed in viable and necrotic regions. Large deposits of Evans-blue were associated with necrotic regions at distance from the infusion site, thus suggesting that necrotic areas could represent preferential pathways (higher $K$) and sinks for the accumulation of therapeutic agents (Figure 2A). The non-uniform distribution of therapeutic agents could significantly limit the success of intratumoral infusions.

To increase $K$, enzymatic degradation of the interstitial matrix could be used. Degradation of the matrix with hyaluronidase increased $K$ by ten-to 20-fold in muscle fascia (Day, 1952), and by a factor of 24 in the lung (Lai Fook et al, 1989). In a preliminary study, we measured $K$ in vivo in two tumours following the intratumoral infusion (0.1 $\mu$l min$^{-1}$) of hyaluronidase. By least squares non-linear regression of the measured pressure profile, the values were $7.7 \times 10^{-7}$ and $11.0 \times 10^{-7}$ cm$^2$/mmHg$^{-1}$ s. These two values are greater than the maximum values of $K$ in the control group (Table 1). Further studies are needed to evaluate the effect of enzyme digestion on $K$ and on the IFP profiles in solid tumours.

In conclusion, we have developed a new technique to estimate $K$ in vivo and after circulatory arrest in tumours with a spherically symmetrical distribution of the fluid infused. The precise spatial resolution of the micropipette technique provides a significant advantage over other techniques for estimating $K$ in vivo. Most importantly the technique can be used to measure and manipulate fluid transport parameters in tumours to improve the delivery of therapeutic agents.

### ACKNOWLEDGEMENTS

We thank Mrs Sylvie Roberge for her technical assistance. The study was supported by an NCI Outstanding Investigator Award (R35 CA56591) to RKJ.

### REFERENCES

Basser PJ (1992) Interstitial pressure, volume, and flow during infusion into brain tissue. *Microvasc Res* **44**: 143–165

Baxter LT and Jain RK (1989) Transport of fluid and macromolecules in tumors. I. Role of interstitial pressure and convection. *Microvasc Res* **37**: 77–104

Bobo HR, Laske DW, Akbasac A, Morrison PF, Dedrick RL and Oldfield EH (1994) Convection enhanced delivery of macromolecules in the brain. *Proc Natl Acad Sci USA* **91**: 2076–2080

Boucher Y and Jain RK (1992) Microvascular pressure is the principal driving force for interstitial hypertension in solid tumors: implications for vascular collapse. *Cancer Res* **52**: 5110–5114

Boucher Y, Baxter L and Jain RK (1990) Interstitial pressure gradients in tissue-isolated and subcutaneous tumors: implications for therapy. *Cancer Res* **50**: 4478–4484

Day TD (1952) The permeability of the interstitial connective tissue and the nature of the interfibrillary substance. *J Physiol, London* **117**: 1–8

DiResta GR, Lee J, Larson SM and Arbit E (1993) Characterization of neuroblastoma xenograft in rat flank. 1. Growth, interstitial fluid pressure and interstitial fluid velocity profiles. *Microvasc Res* **46**: 158–177

Fatt I (1968) Dynamics of water transport in the corneal stroma. *Exp Eye Res* **7**: 402–412

Ford TR, Sachs JR, Grotberg JB and Glucksberg MR (1991) Perialveolar interstitial resistance and compliance in isolated rat lung. *J Appl Physiol* **70**: 2750–2756

Guyton AC, Scheel K and Murphree D (1966) Interstitial fluid pressure: its effect on resistance to tissue fluid mobility. *Circ Res* **19**: 412–419

Heise C, Sampson-Johannes A, Williams A, McCormick F, Von Hoff DD and Kirn DH (1997) ONYX-O15, an EIB gene-attenuated adenovirus, causes tumor-specific cytolysis and antitumoral efficacy that can be augmented by standard chemotherapeutic agents. *Nature Med* **3**: 639–645

Jain RK (1987) Transport of molecules in the tumor interstitium: a review. *Cancer Res* **47**: 3038-3050

Jain RK (1993) Physiological resistance to the treatment of solid tumors. In *Drug Resistance in Oncology*, BA Teicher (ed.), pp. 87–105. Marcel Dekker: New York

Jain RK and Baxter LT (1988) Mechanisms of heterogeneous distribution of monoclonal antibodies and other macromolecules in tumors: significance of elevated interstitial pressure. *Cancer Res* **48**: 7022–7032

Jirtle RL (1988) Chemical modification of tumour blood flow. *Int J Hyperthermia* **4**: 355-371

Lai Fook, SJ, Rochester NL and Brown LV (1989) Effects of albumin, dextran and hyaluronidase on pulmonary interstitial conductivity. *J Appl Physiol* **67**: 606–613

Levick JR (1979) The influence of hydrostatic pressure on trans-synovial fluid movement and on capsular expansion in the rabbit knee. *J Physiol* **289**: 69–82

Levick JR (1980) Contributions of the lymphatic and microvascular systems to fluid absorption from the synovial cavity of the rabbit knee. *J Physiol* **306**: 445–461

Levick JR (1987) Flow through interstitium and other fibrous matrices. *Q J Exp Physiol* **72**: 409–438

Mellander S (1960) Comparative studies on the andrenergic neuro-hormonal control of resistance and capacitance blood vessels in the cat. *Acta Physiol Scand* **50** (suppl. 176): 1–86

Morrison PF, Laske DW, Bobo H, Oldfield EH and Dedrick RL (1994) High-flow microinfusion:tissue penetration and pharmacodynamics. *Am J Physiol* **266**: R292–R305

Netti PA, Baxter LT, Boucher Y, Skalak R and Jain RK (1995) Time-dependent behavior of interstitial fluid pressure in solid tumors: implication for drug delivery. *Cancer Res* **55**: 5451–5458

Nomura T, Nakajima S, Kawabata K, Yamashita F, Takakura Y and Hashida M (1997) Intratumoral pharmacokinetics and in vivo gene expression of naked plasmid DNA and its cationic liposome complexes after direct gene transfer. *Cancer Res* **57**: 2681–2686

Order SE, Siegel JA, Lustig RA, Principato R, Zeiger LS, Johnson E, Zhang H, Lang P and Wallner PE (1994) Infusional brachytherapy in the treatment of non-resectable pancreatic cancer: a new radiation modality (preliminary report of the phase I study). *Immunoconj Radiopharm* **7**: 11–27

Sands H (1992) Radiolabeled monoclonal antibodies for cancer therapy and diagnosis: is it really a chimera? *J Nucl Med* **33**: 29–32

Sevick EM and Jain RK (1991) Measurement of capillary filtration coefficient in a solid tumor. *Cancer Res* **51**: 1352–1355

Swabb EA, Wei J and Gullino PM (1974) Diffusion and convection in normal and neoplastic tissues. *Cancer Res* **34**: 2814–2822

Thompson WD, Shiach KJ, Fraser RA, McIntosh LC and Simpson JG (1987) Tumours acquire their vasculature by vessel incorporation, not vessel ingrowth. *J Pathol* **151**: 323–332

Tokita M and Tanaka T (1991) Friction coefficient of polymer networks of gels. *J Chem Phys* **95**: 4613–4618

Tozer GM, Lewis S, Michalowski A and Aber V (1990) The relationship between regional variations in blood flow and histology in a transplanted rat fibrosarcoma. *Br J Cancer* **61**: 250–257

Viola JJ, Agbaria R, Walbridge S, Oshiro EM, Johns DG, Kelley JA, Oldfield EH and Ram Z (1995) *In situ* cyclopentenyl cytosine infusion for the treatment of experimental brain tumors. *Cancer Res* **55**: 1306–1309

Zawieja DC, Garcia C and Granger HJ (1992) Oxygen radicals, enzymes, and fluid transport through pericardial interstitium. *Am J Physiol* **262**: H136–H143

 © Cancer Research Campaign 1998